# Characterization of human FDCs reveals regulation of T cells and antigen presentation to B cells

**DOI:** 10.1084/jem.20210790

**Published:** 2021-08-23

**Authors:** Balthasar A. Heesters, Kyah van Megesen, Ilhan Tomris, Robert P. de Vries, Giuliana Magri, Hergen Spits

**Affiliations:** 1 Amsterdam University Medical Centers, University of Amsterdam, Department of Experimental Immunology, Amsterdam institute for Infection and Immunity, Amsterdam, Netherlands; 2 Department of Chemical Biology and Drug Discovery, Utrecht Institute for Pharmaceutical Sciences, Utrecht University, Utrecht, Netherlands; 3 Program for Inflammatory and Cardiovascular Disorders, Institut Hospital del Mar d’Investigacions Mèdiques, Barcelona, Spain

## Abstract

Stromal-derived follicular dendritic cells (FDCs) are essential for germinal centers (GCs), the site where B cells maturate their antibodies. FDCs present native antigen to B cells and maintain a CXCL13 gradient to form the B cell follicle. Yet despite their essential role, the transcriptome of human FDCs remains undefined. Using single-cell RNA sequencing and microarray, we provided the transcriptome of these enigmatic cells as a comprehensive resource. Key genes were validated by flow cytometry and microscopy. Surprisingly, marginal reticular cells (MRCs) rather than FDCs expressed B cell activating factor (BAFF). Furthermore, we found that human FDCs expressed TLR4 and can alter antigen availability in response to pathogen-associated molecular patterns (PAMPs). High expression of PD-L1 and PD-L2 on FDCs activated PD1 on T cells. In addition, we found expression of genes related to T cell regulation, such as *HLA-DRA*, *CD40*, and others. These data suggest intimate contact between human FDCs and T cells.

## Teaser

Single-cell RNA sequencing of human follicular dendritic cells (FDCs) provides insights in germinal center biology and enhances our understanding of these enigmatic cells. Data suggest regulation of B cell antigen availability by FDCs and T cell interactions with FDCs.

## Introduction

The germinal center (GC) structure within B cell follicles is crucial for affinity maturation, which generates effective antibodies. Within the light zone of the GC and in resting B cell follicles, a few follicular dendritic cells (FDCs) reside. These poorly understood cells were discovered in 1964 as antigen-retaining reticular cells. FDCs are the archetype stromal cell of the B cell follicle and, as the name suggests, have many dendrites and can retain antigen (Nossal et al., 1964). FDCs are of mesenchymal origin and are thus unrelated to classic dendritic cells. Together with marginal reticular cells (MRCs), fibroblastic reticular cells (FRCs), and other lymph node stromal cells, FDCs form a continuous network that functions as a backdrop for immune responses. MRCs are located against the subcapsular sinus and extend into the interfollicular region, almost forming a ring-like structure around the B cell follicle, and are thought to be involved in tissue development and antigen capture. The most dominant stromal subsets in the T cell area, characterized by podoplanin (PDPN) expression, are the FRCs (Krishnamurty and Turley, 2020).

FDCs form the scaffold of the GC by secretion of CXCL13 (Ansel et al., 2000; Tew et al., 1990). The CXCL13 chemokine gradient attracts CXCR5^+^ cells, such as B cells and follicular T cells, effectively forming the B cell follicle. The dendritic phenotype of the FDC enables intimate contact with surrounding cells. The most striking property that sets FDCs apart from other cells is the ability to retain native antigen for a long time and present it to B cells in the GC to aid B cell selection (Hanna and Szakal, 1968; Heesters and Carroll, 2016; Heesters et al., 2013; Mandels et al., 1980; Nossal et al., 1968). Yet FDCs are not passive antigen depots and use protective endosomal compartments to periodically cycle antigen to their surface (Heesters et al., 2013). Antigen remains intact as stable complement opsonized immune complexes (ICs) and can be presented in its native form to B cells (Heesters et al., 2016; El Shikh and Pitzalis, 2012; Heesters et al., 2013). The context of antigen presentation is essential and suggests a more regulatory role for FDCs, for instance, by regulation of antigen availability or through Fc receptor engagement or other signals (Arulraj et al., 2021; van der Poel et al., 2019). Little is known about coreceptors or other signaling molecules undoubtedly involved in the interactions between FDCs and lymphocytes.

Most functional studies are performed in mice and evaluated by immunohistochemistry or indirect readouts (e.g., serum antibody levels). The data available on human FDCs are based almost exclusively on micrographic evidence, and as a result, FDCs are identified by their extensive dendritic morphology and a limited number of markers, such as complement receptor (CR) 1, CR2, milk fat globule–EGF factor 8 (MFGE8), and vascular cell adhesion molecule 1 (VCAM1). The advance in sequencing techniques has brought transcriptomics of FDCs within reach, and indeed, recent elegant studies of lymph node stromal cells in mice included some FDCs in their analyses (Pikor et al., 2020; Rodda et al., 2018; Suzuki et al., 2010). In this study, we aimed to unravel the human FDC transcriptome and focused on the function of human FDCs in the GC microenvironment. The transcriptomic data presented here uncovered new insights and revealed expression of many sensory and immunomodulatory molecules. Functional experiments showed TLR4 activation on FDCs to increase antigen presentation to specific B cells. Studies in mice support this data and showed TLR4 expression on FDCs to be linked to GC formation, FDC activation, and high-affinity antibody production (El Shikh et al., 2007; Garin et al., 2010). Furthermore, the unexpected expression of immune modulatory molecules programmed death (PD) ligand 1 (CD274, PD-L1) and PD1 ligand 2 (PDCD1LG2, PD-L2) on FDCs was functional as it activated PD1 on T cells in vitro. This suggested a role for FDCs in B cell and T cell regulation in the GC.

Finally, FDCs are scarce and localize together in highly interconnected three-dimensional networks within B cell follicles, which makes them very difficult to isolate and culture. The challenge to study FDCs has resulted in a void of knowledge about these cells that play such a central role in adaptive immunity. Combined, our experiments fill this void and suggest a regulatory role for FDCs in adaptive immune responses in the GC for B cells and T cells.

## Results

### Transcriptome analysis of human FDCs

**Figure S1.**
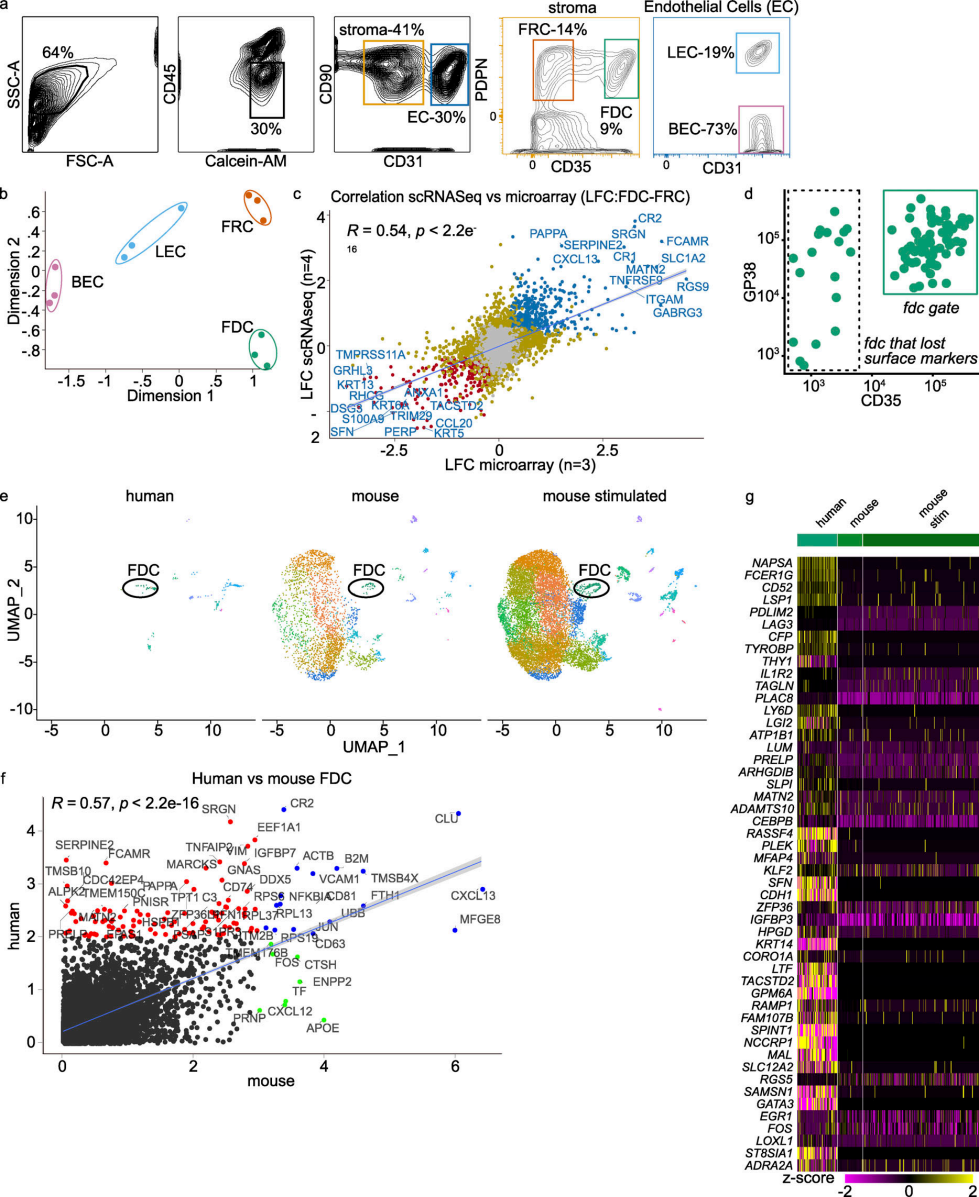
**scRNaseq, microarray, and scRNaseq mouse transcriptome data correlate well.** **(a)** Gating strategy for microarray. CD45^−^, live cells were gated for stroma and endothelial cells. Within the stroma gate, a gate for FRCs (PDPN^+^CD35^−^) and FDCs (PDPN^+^CD35^+^) was set, while in the endothelial gate, lymph endothelial cells (LECs; PDPN^+^CD31^+^) and blood endothelial cells (BECs; PDPN-CD31^+^) were defined. **(b)** Multi-Dimensional Scaling plot shows clustering of the microarray samples. Dimensions 1 and 2 account for 74.1% and 12.3% of variance, respectively. **(c)** Correlation of log fold change of FDCs versus FRCs between scRNaseq and microarray. Top genes were labeled. **(d)** FACS plot on FDC cluster through index sort. Most cells from the FDC cluster fall within the FDC gate. Minimal cleavage of extracellular markers by digestive enzymes. GP38 (PDPN) versus CD35 (CR1). **(e)** Uniform Manifold Approximation and Projection (UMAP) clustering of integrated datasets after conversion of mouse genes to human orthologues (one-on-one) and discarding nonmatching genes. FDC cluster is indicated. Mouse scRNaseq data from Rodda et al. (2018) (GEO accession no. GSE112903). **(f)** Mean expression of human versus mouse FDCs. Genes with no expression in either dataset were ignored. Red indicates top genes expressed more in human, blue indicates top genes in agreement, and green indicates top genes expressed more in mouse. **(g)** Top 50 most differentially expressed genes mouse versus human. scRNaseq violin plot on major clusters. scRNaseq data, *n* = 4; microarray data *n* = 3 (biological replicates). FSC-A, forward scatter area; SSC-A, side scatter area; stim, stimulated.

**Figure 1.**
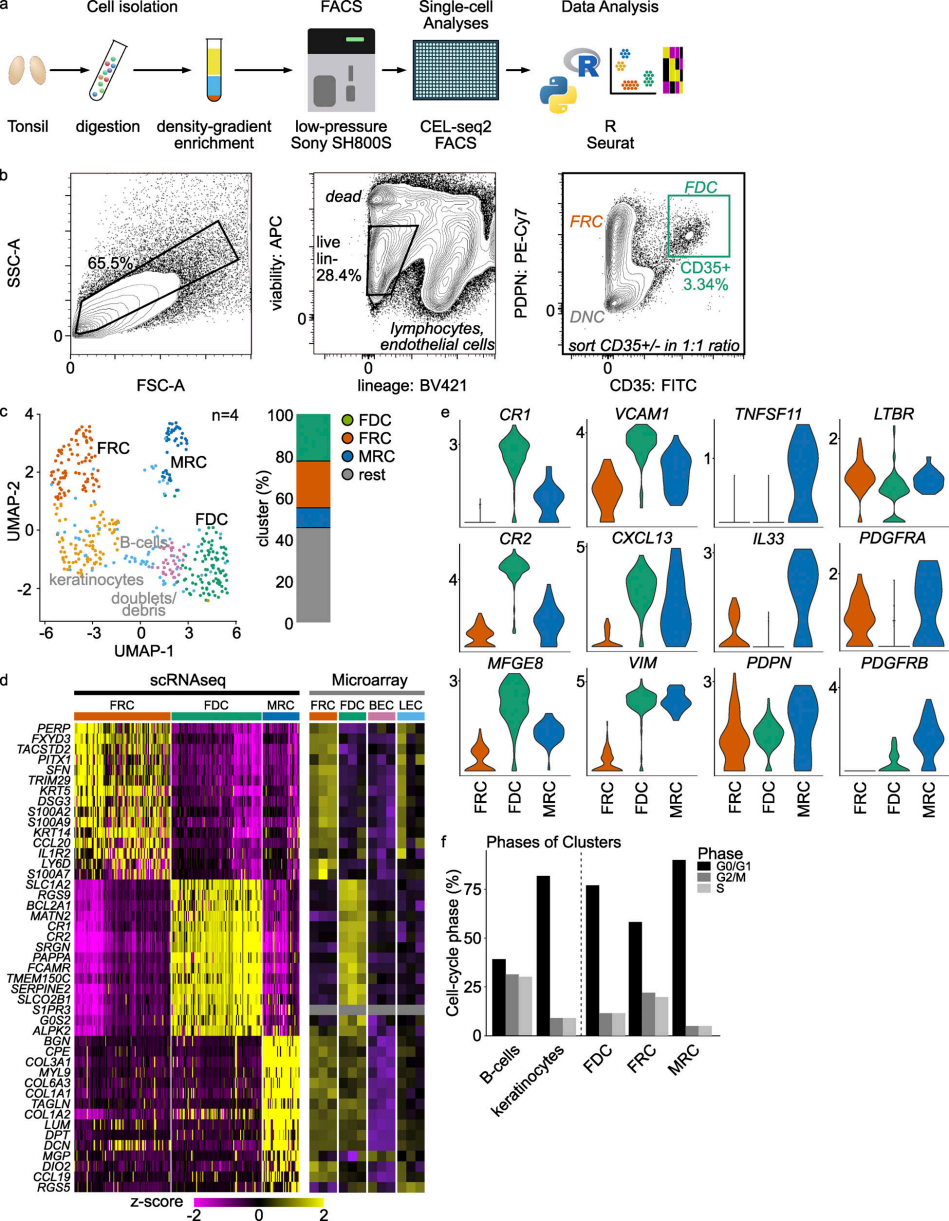
**Single-cell RNA sequencing of FDCs: clusters and validation.** **(a)** Schematic of the scRNaseq workflow. Tonsils were gently digested to a single-cell suspension, enriched for FDCs by Percoll gradient centrifugation, sorted in 384-well CEL-seq2 plates, and aligned and analyzed in python and R. **(b)** Gating strategy. SSC-A^high^ cells were selected, then live CD45^−^CD31^−^ cells. From here, FDCs were defined as PDPN^+^CD35^+^. **(c)** Unbiased Uniform Manifold Approximation and Projection (UMAP) clustering was used to determine similar cell types; doublets, B cells, and keratinocytes were removed. **(d)** Heatmap of most differentiating genes in scRNaseq with the same genes in the microarray. Yellow is high expression; purple is low expression. **(e)** Known genes for the cell populations of interest. Complement receptors and *CXCL13* set the FDCs apart, while *TNFSF11* (*RANKL*) and *IL-33* define MRC. FRCs were *PDPN*^*+*^, *LTBR*^*+*^, and *PDGFRA*^*+*^, as expected. Sctransform-normalized expression. **(f)** Cell cycle phases of the clusters as determined by G0-, G1-, and S-phase genes. Related to Fig. S1 and Fig. S2. scRNaseq data, *n* = 4; microarray data, *n* = 3 (biological replicates). BEC, blood endothelial cell; FSC-A, forward scatter area; LEC, lymph endothelial cell; SSC-A, side scatter area.

To determine the regulatory proteins expressed by human FDCs, we decided to perform a transcriptome analysis on sorted FDCs from tonsillar tissues. The tonsil is one of the few human tissues routinely available that has ongoing GCs. In our optimized isolation protocol, FDCs comprised only 1–4% of stromal cells in the tonsil after a Percoll gradient (Heesters et al., 2017). We started with bulk transcriptome analysis of FDCs and other stromal cells by microarray (Fig. S1, a–c). This analysis showed distinct clustering of the sorted cell types (FDCs, FRCs, lymph endothelial cells, and blood endothelial cells) and provided insights into their biology (Fig. 1 d and Fig. S1 b). However, bulk transcriptome analysis cannot determine if expression is reflective of a single, homogenous cell population or the average of a heterogeneous, perhaps mixed, population. To resolve this, we used single-cell RNA sequencing (scRNaseq) on human tonsil stromal cells enriched for FDCs (Fig. 1 a). Indeed, this provided populations of higher purity, although the two datasets showed high correlation (Fig. S1 c). Tonsils from four donors were digested, filtered, and enriched for stromal cells with a Percoll gradient to obtain a single cell solution. Then, stromal cells were sorted into 384-well plates, sequenced by CELSeq2, and subsequently analyzed (Fig. 1 a). To enrich for FDCs, we sorted CD45^−^CD31^−^PDPN^+^CD35^+^ (FDCs) and CD45^−^CD31^−^CD35^−^ (nonlymphocytes, nonendothelial cells, non-FDCs) in a one-to-one ratio (Fig. 1 b). Initial unsupervised clustering revealed six clusters, and after selection for stromal, live, and single cells, three clusters remained (Fig. 1 c). We identified these clusters as FRCs, FDCs, and MRCs based on the expression of marker genes (Fig. 1, d and e). The FDC cluster highly expressed the classic FDC marker genes *CR1*, *CR2*, *MFGE8*, *VCAM1*, and *CXCL13* (van Nierop and de Groot, 2002). MRCs expressed high levels of *TNFSF11* (*RANKL*) and *IL33*, while FRCs expressed *PDPN*, lymphotoxin β receptor (*LTBR*), *CCL20*, and platelet-derived growth factor receptor α (*PDGFRA*), as expected (Katakai, 2012; Malhotra et al., 2013). FDCs were negative for *PDGFRA* and positive for platelet-derived growth factor receptor β (*PDGFRB*), while FRCs and MRCs were positive for *PDGFRA*. In the microarray, both FRCs and FDCs were positive for *PDGFRA* and *PDGFRB*. This difference can be due to differences in sensitivity between the techniques. The expression of *PDGFRB* by FDCs and the expression of *PDGFRA* by FRCs was expected (Cheng et al., 2019; Fletcher et al., 2015).

We compared the human scRNaseq data with scRNaseq data generated in mice by one-to-one conversion of mouse genes to human orthologues (Rodda et al., 2018). Integration of these datasets served two purposes, additional validation and exploration of mouse–human differences in FDCs (Fig. S1 e). Most importantly, the human FDC transcriptome correlated well with the mouse FDC transcriptome (Fig. S1, f and g). Key genes that define FDCs, such as *CR2*, *CXCL13*, *MFGE8*, and *VCAM1*, were present in both datasets. *SERPINE2*, *TMSB10*, and* PRELP* were genes that stood out in the human data and are all involved in extracellular matrix organization. These differences can be due to the location of the lymphoid tissue or species. To make definitive conclusions, FDCs from different species but similar anatomical locations should be compared.

Analysis of cell cycle phase indicated that human FDCs mainly resided in the G0/G1 phase, as expected (Fig. 1 f). This was the same for FRCs and MRCs, cell subsets that propagate well in culture (Fletcher et al., 2011; Katakai et al., 2004). The human scRNaseq FDC cluster data correlated well with the human microarray data on FDCs (Fig. S1 c). Although index sorting revealed the FDC cluster to predominantly stain positive for PDPN (GP38) and CD35 protein on the surface, some FDCs had lost these markers during the isolation protocol (Fig. S1 d). Therefore, it is of the utmost importance to digest the tissue as gently as possible.

### FDC surface markers

**Figure 2.**
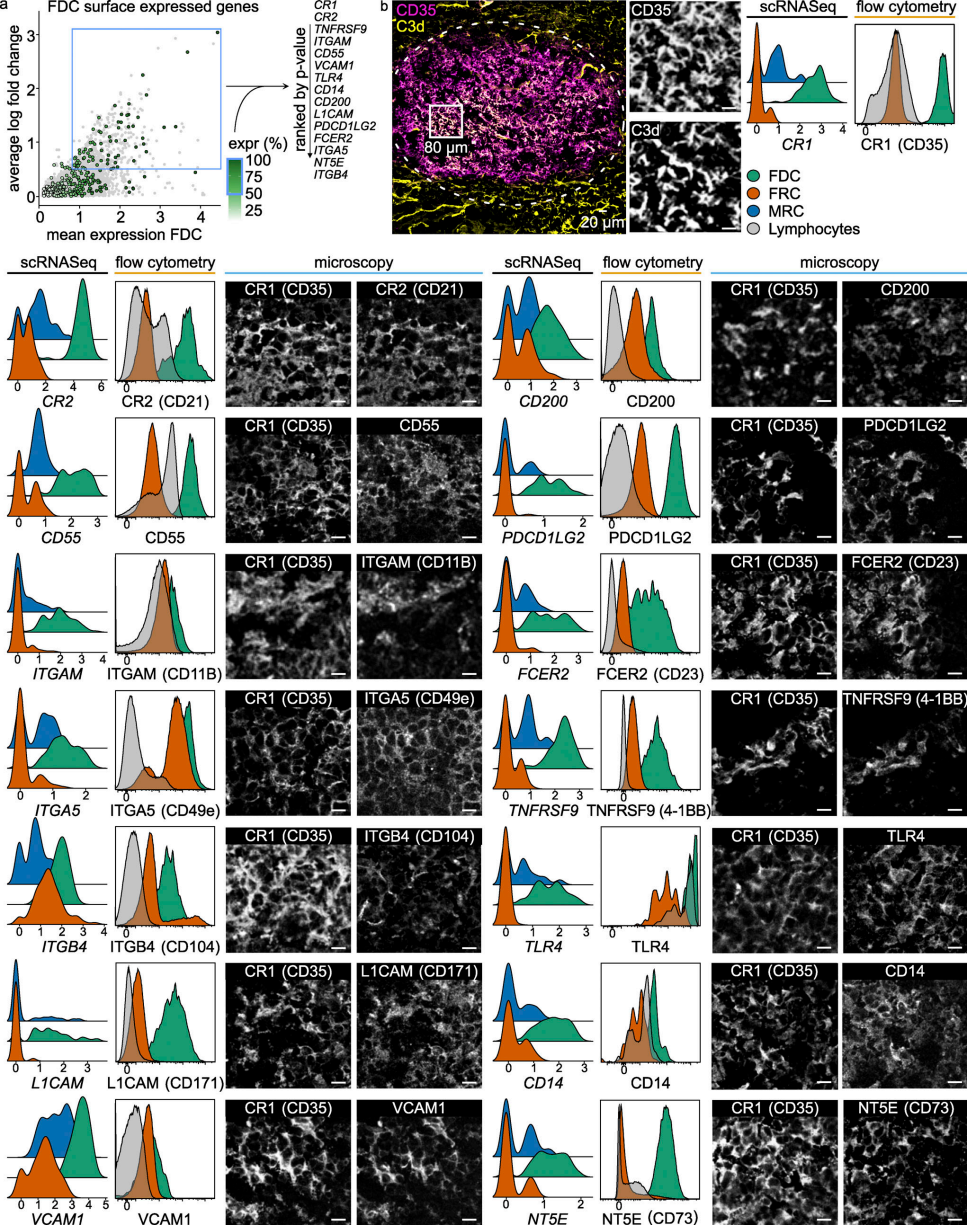
**FDC surface marker expression.** **(a)** Mean gene expression of FDCs versus the log fold change against FRCs and MRCs of surface expressed proteins. Hue indicates percentage of FDCs with expression. List ranks top 15 genes by P value. Blue squares indicate selection criteria. **(b)** scRNaseq data alongside flow cytometry and microscopy data. Cryopreserved tonsil section stained for CR1 and C3d to identify FDCs. Scale bar, 20 µm. Inserts are 40-µm-sided squares with a 10-µm scale bar. Dotted line indicates follicle. scRNaseq data show FDC, FRC, and MRC expression of the indicated gene. Flow cytometry data show FDC, FRC, and lymphocyte surface expression of indicated proteins. MRCs were not included in flow cytometry. Populations gated as CD45^−^CD31^−^PDPN^+^CD35^+^, CD45^−^CD31^−^PDPN^+^CD35^−^, and CD45^+^CD31^−^, respectively. Microscopy inserts are from a 80-µm by 80-µm area within the follicle. Left: CD35. Right: The indicated protein of interest. TLR4 microscopic image is divided to show results with and without a boost in gain level. Sctransform-normalized expression. Scale bar, 10 µm. Pearson’s colocalization is shown in Fig. S3 d. scRNaseq data, *n* = 4; microscopy and flow cytometry data, *n* > 3 (biological replicates). expr, expression.

**Figure S2.**
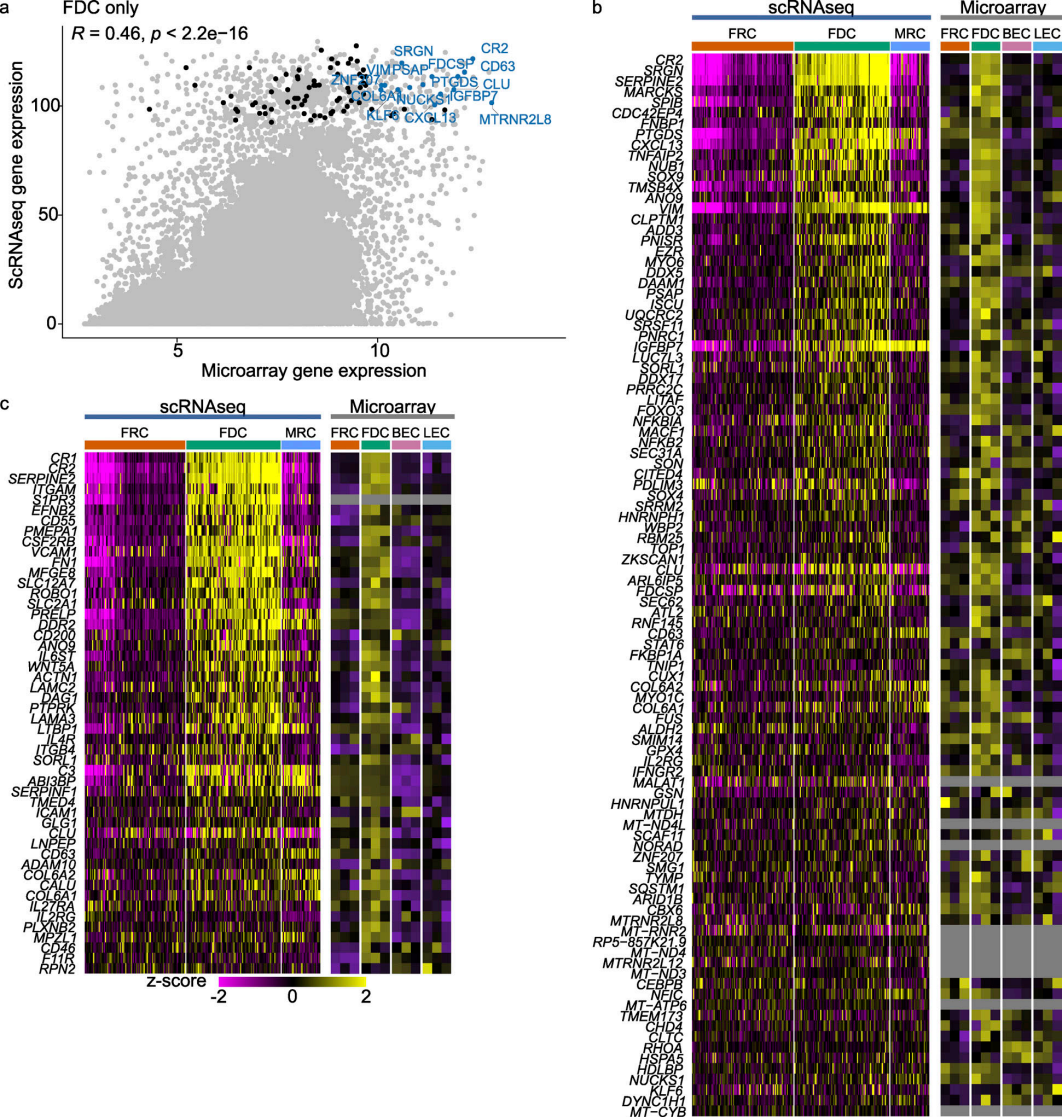
**Top FDC genes.** **(a)** Average expression scRNaseq versus average expression microarray. Top 100 highest expressed genes in >90% of FDCs by scRNaseq are labeled as black dots; top 15 genes are labeled. R is correlation of scRNaseq and microarray FDC groups. **(b)** Top 100 FDC genes from (a) as heatmaps of scRNaseq and microarray. **(c)** Top 50 surface-expressed FDC genes (according to the human surface protein atlas) with corresponding heatmaps from scRNaseq and microarray. scRNaseq data, *n* = 4; microarray data, *n* = 3 (biological replicates). BEC, blood endothelial cell; LEC, lymph endothelial cell.

**Figure S3.**
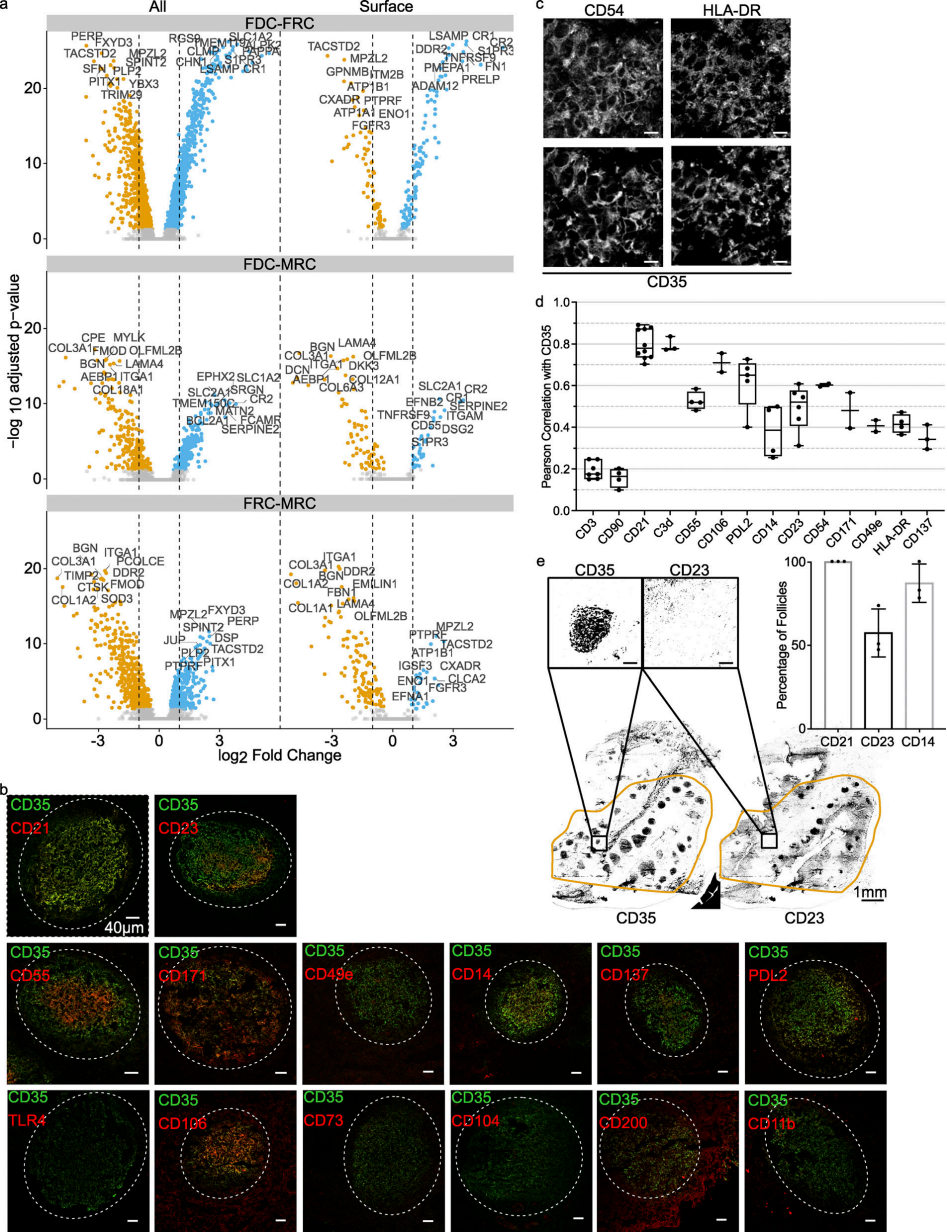
**FDC volcano plots and microscopy.** **(a)** Volcano plots of different cluster combinations. Top 10 genes are labeled. Significant differential expressed genes are colored orange (down) or blue (up). Dotted line indicates twofold log change. **(b)** Images of the full follicle to assess outside follicle background. Scale bars, 40 µm. **(c)** Additional fluorescent microscopy staining of human tonsil sections. Scale bar, 10 µm. **(d)** Pearson’s correlation of markers with CD35 by microscopy. Quantification corresponds to Fig. 2 c. Within a follicle region of interest, the colocalization between CD35 and the corresponding marker was calculated by Pearson’s correlation. **(e)** Percentage of follicles with CD14, CD23, or CD21. After thresholding, positive and negative follicles were counted by hand. Example of CD23 tonsil slice after threshold. Scale bars, 1 mm; insert, 100 µm. scRNaseq data, *n* = 4; microscopy data, *n* = 3 (biological replicates); Pearson’s correlation, *n* > 3 (biological replicates).

The surfactome of FDCs is not well established because FDCs are not regularly analyzed by flow cytometry like most hematopoietic immune cells. Here, scRNaseq data were filtered to find proteins expressed on the cell membrane of >50% of cells with high expression levels on FDCs and minimal expression by other stromal cells (Fig. 2 a). Genes were selected for suspected protein surface expression based on the human surface protein atlas (Bausch-Fluck et al., 2015). Heatmaps of all top genes gave insights into the relationship of FDCs with other subsets and the microarray data (Fig. S2, a–c; and Fig. S3 a). As expected, CD35 (CR1) identified FDCs well by tissue immunofluorescence microscopy and flow cytometry. CD35 localized together with C3d, which identified ICs, in a typical stellate dendritic network (Fig. 2 b). Selected genes were validated by flow cytometry, which in general showed a consistent pattern of expression (Fig. 2 b). As most of the protein expression characterization in FDCs was based on tissue staining, we validated our findings by immunofluorescence microscopy. This analysis confirmed the co-expression of several of these molecules, while others could not be detected as well, probably due to the disruption of epitopes upon tissue fixation (Fig. S3, b and c). Co-localization with CD35 was determined by Pearson’s correlation (Fig. S3 d). Unexpectedly, differential expression of CD23 and CD14 was observed between follicles within the same tonsil (Fig. S3 e). As many macrophages and B cells reside in the follicle in close contact with the FDCs, microscopy on shared markers is unreliable. Therefore, scRNaseq was leading in the selection of genes. The known functions of these highly expressed genes are briefly discussed below. Classical FDC markers *CR1* and *CR2* are receptors that capture antigen opsonized with complement. High gene expression of receptors was not necessarily expected since they cycle through nondegradative compartments and protein turnover could be low (Heesters et al., 2013). This proved not to be the case; CR1 (CD35) and CR2 (CD21) were reliable markers for FDCs at the gene and protein levels. Another highly expressed complement gene was CD55, which blocks the propagation of the complement cascade. This is useful to retain ICs without further cleavage of complement components. Furthermore, FDCs express ITGAM (CD11B), also known as CR3, which can bind complement iC3b. Other members of the integrin family were expressed as well. ITGA5 (CD49e) binds fibronectin, probably anchoring FDCs and FRCs to the extracellular matrix. ITGB4 has a similar function by binding to collagen. Adhesion can also be facilitated by L1CAM, a cell adhesion molecule involved in motility and neuronal development. Expression of VCAM1 on FDCs facilitates adhesion of lymphocytes. FDCs expressed various molecules that can regulate lymphocytes and other immune cells, such as CD200, which can inhibit immune cells through CD200R, for instance, to inhibit macrophage activity at the FDC surface, where antigen is present. Unexpectedly, the PD1 ligand PDCD1LG2, better known as PD-L2, was highly expressed by FDCs. The role of the many fragment crystallizable region receptors (FcRs) expressed by FDCs is still unclear. FCER2 (CD23), the low-affinity receptor for IgE, was highly expressed and is known for its role in antibody feedback regulation in B cells. TNFRSF9 (4-1BB) expression by FDCs was unexpected. 4-1BB can activate NF-κB and signal from within endosomes. The ligand for 4-1BB (4-1BBL) is expressed by GC B cells. LPS can activate TLR4, which forms a complex with CD14 and allows for detection of bacteria by FDCs. NT5E is an ecto-5-prime-nucleotidase that mediates the gradual hydrolysis of the (autocrine and paracrine) danger signals ATP and ADP to the anti-inflammatory adenosine.

### Pathway analysis

**Figure 3.**
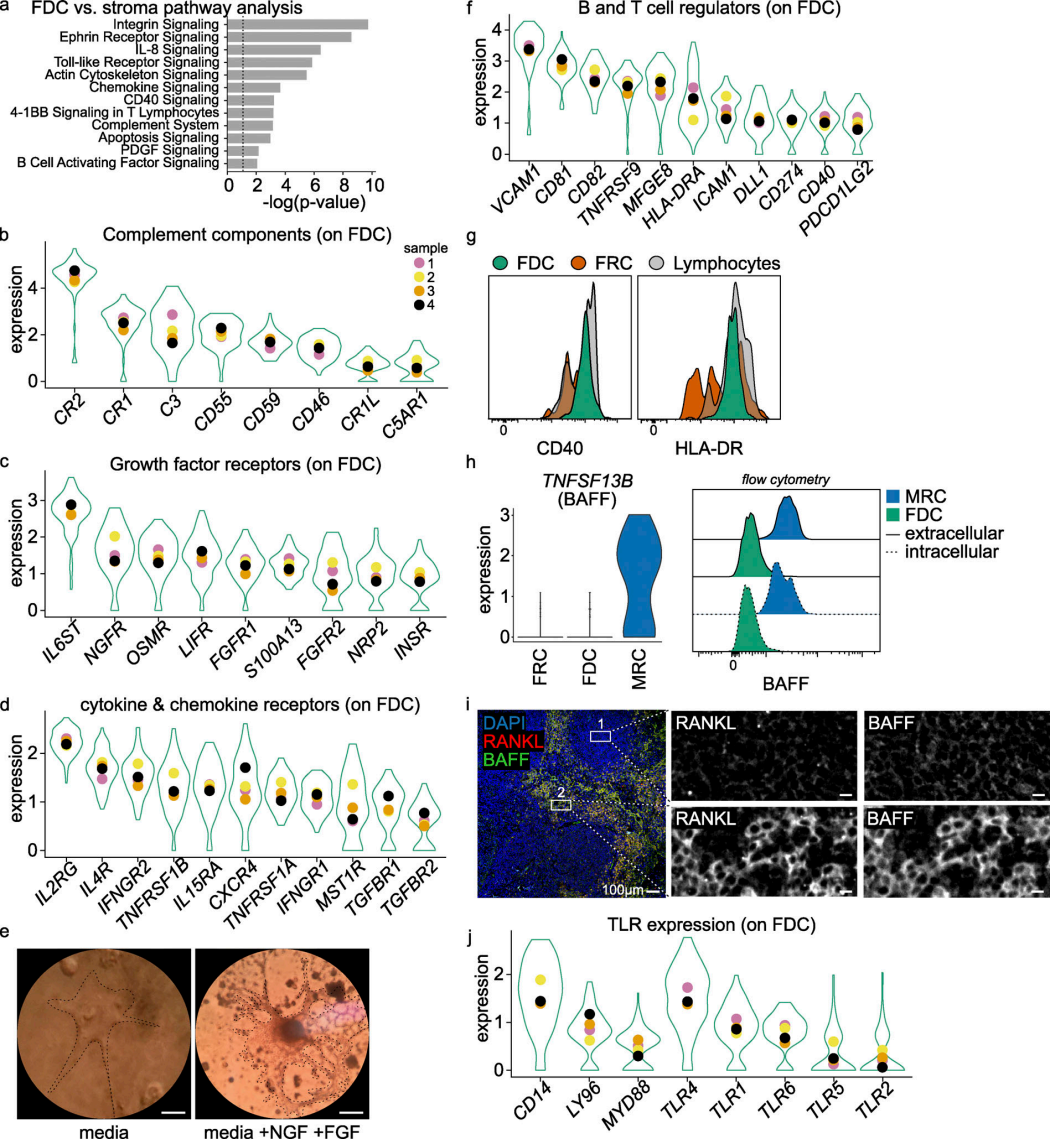
**FDC key pathways.** **(a)** Ingenuity pathway analysis was performed on differential expressed genes of the FDC dataset compared with the FRC and MRC datasets. The most significant pathways are listed. **(b–d, f, and h) **Most expressed pathway gene expression in FDCs. Violin plot is aggregate data, and colored dots are mean value per donor. All genes were above expression threshold. **(e)** Micrograph of sorted FDCs after 7-d culture in media with NGF and FGF. Scale bar, 10 µm. **(g)** CD40 and HLA-DR fresh protein expression on FDCs compared with FRCs and lymphocytes. **(i)** BAFF expression in FDCs, FRCs, and MRCs by scRNaseq and BAFF protein expression in FDCs and MRCs by flow cytometry. Intra- and extracellular staining. **(j)** Immune fluorescent image of tonsil section. Scale bar, 100 µm. Box 1 is central to the FDC area, and box 2 MRCs are stained by RANKL. Scale bar, boxes, 10 µm. scRNaseq data, *n* = 4 (biological replicates), Sctransform-normalized expression. Related to Fig. S3 and Fig. S4 a.

**Figure S4.**
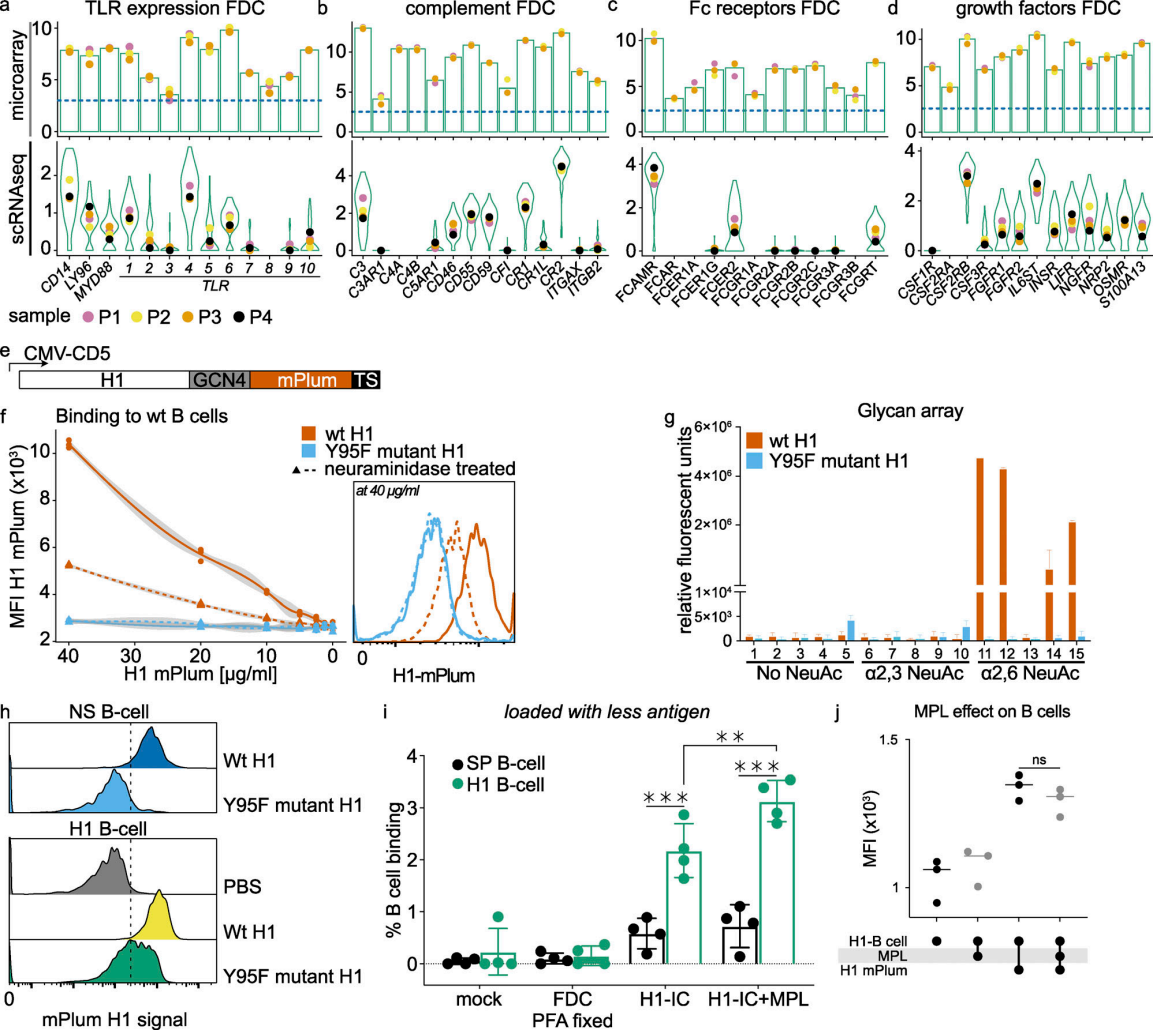
**FDC genes microarray and hemagglutinin controls.** **(a)** Expression of TLRs on FDCs by microarray and scRNaseq. *n* = 3 and *n* = 4, respectively (biological replicates). Bars represent average expression, and violin plots aggregate all cells. Colored dots indicate (average) expression of donors. **(b)** As in (a) for complement components. **(c)** As in (a) for Fc receptors. **(d)** As in (a) for growth factors. **(e)** Glycoprotein expression plasmid with GCN4 trimerization motif with C-terminal mPlum. Schematic representation of used expression system with CMV promoter. The H1 open reading frame was cloned in frame with the CD5 signal peptide, followed by a GCN4 trimerization motif, mPlum, and a TEV cleavable twin-strep-tag at its C terminus. **(f)** Binding of H1-mPlum to WT B cells (Raji), which express α2,6 NeuAc. MFI of mPlum on B cells was measured at different concentrations of H1-mPlum. Binding is neuraminidase-sensitive and dose-dependent; the Y95F mutant does not bind. Flow cytometry histogram is shown at the highest concentration (40 µg/ml H1-mPlum). **(g)** Glycan array, WT H1-mPlum (PR8) binds to α2,6 NeuAc moieties while the Y95F H1-mPlum mutant (PR8) does not. Exact composition of glycans can be found in Table 1. **(h)** Y95F mutant binding to specific (H1 B cell) and NS-B cells (*S. pneumoniae*–specific) compared with WT H1 binding. Residual binding on NS-B cells disappears with the mutant. **(i)** Percentage H1-mPlum–positive B cells after incubation with FDCs loaded with 100-fold less H1-IC as in Fig. 4; *n* = 4 (biological replicates). H1-B cell (influenza H1–specific) and SP-B cell (*S. pneumoniae*–specific). MPL (TLR4 agonist). **(j)** Effect of MPL on H1-mPlum binding capability of H1–B cells. B cells were incubated with 100 ng/ml MPL, and H1-mPlum was added. No difference in binding was reported. *n* = 3 (biological replicates). Student’s *t* test or ANOVA, **, P > 0.01; ***, P > 0.001. Related to Table 1. TS, thymidylate synthase promotor.

To get information on the functionality of expressed gene clusters, an ingenuity pathway analysis was performed on differentially expressed genes in FDCs compared with MRCs and FRCs. This analysis revealed expected pathways, such as the complement cascade, actin cytoskeleton signaling, and B cell interactions. TLR signaling, various T cell interaction pathways, and cell survival signaling were found as well (Fig. 3 a), suggesting that FDCs interact not only with B cells but also with T cells. Genes for these pathways were also detected in the microarray data (Fig. S4 a).

### Complement components in FDCs

As expected, FDCs expressed many complement components involved in IC binding, such as *CR1*, *CR2*, and *CR1L*. These complement receptors are hallmark FDC markers and are widely used for identification of FDCs (Fig. 3 b and Fig. S4 b). CD55, which prevents the formation of C3- and C5-convertases, was also highly expressed by FDCs (Fig. 3 b; Fujita et al., 1987). Furthermore, the expression of *C5AR1* (CD88), the receptor for the anaphylatoxin C5a, provides a possibility for the FDCs to sense the inflammatory state of the environment and the amount of complement activation (Fig. 3 b). The expression of complement *C3* by FDCs was unexpected since it is generally assumed that most soluble C3 is produced in the liver, and local production is subscribed to macrophages (Alper et al., 1969; Fischer et al., 1998).

### Expression of growth and survival factors and their receptors

The ability to culture sorted human FDCs would facilitate functional studies. Therefore, we analyzed expression of growth and survival factors potentially required for FDC maintenance. We observed expression of several fibroblast growth factor (FGF) receptors (*FGFR1*, *FGFR2*, and *S100A13*), the VEGF receptor *NRP2*, insulin receptor (*INSR*), and nerve growth factor (NGF) receptor (*NGFR*). In addition, expression of *IL6ST*, *LIFR*, and *OSMR* was observed (Fig. 3 c and Fig. S4 d). IL-6ST can form a complex with many receptors, such as type I cytokine receptors, leukemia inhibitory factor receptor (LIFR), or oncostatin M receptor (OSMR). Signaling through these receptors is complex but appears to influence cell differentiation. Chemokines and cytokines could provide signals as well. IL-4R can combine with the common γ chain (IL-2RG) to form the IL-4 receptor or with IL-13RA to form the IL-13 receptor. FDCs expressed *IL4R* and *IL2RG* at high levels and thus likely respond to IL-4 (Fig. 3 d). Response to IFN-γ by FDCs is also expected since the IFN-γ receptor is formed by subunits *IFNGR2* and *IFNGR1*, which are both expressed by FDCs (Fig. 3 d). Expression of *TGFBR1*, *TGFRB2*, *TNFRSF1B*, and *TNFRSF1A* indicated possible responses to TGF-β and TNF-α, respectively. These insights provided a rationale to modify the FDC growth media, and indeed, supplementation with FGF and NGF improved FDC cultures after flow-cytometric cell sorting (Fig. 3 e). FDCs were more numerous and had a more dendritic phenotype compared with cultures not supplemented with FGF and NGF.

### FDCs express molecules to interact with B and T cells

CXCL13 secreted by FDCs attracts not only B cells but T follicular cells as well, both through CXCR5. T follicular helper (TFH) cells are important during B cell selection in the primary and secondary GCs (Mesin et al., 2020; Ritvo et al., 2018; Zhang et al., 2018). As expected, we found molecules capable of interaction with B cells in our data, such as *CD81*, *CD82*, and *VCAM1* (Fig. 3 f). Also, expression of *DLL1*, *CD40*, and *HLA-DRA* together with related genes such as *CD74* suggests intimate contact between FDCs and T cells. FRCs can express *HLA-DRA* as well, which is up-regulated in response to IFN-γ in vitro (Baptista et al., 2014). In addition, our data showed expression of many other T cell regulatory proteins, such as *CD274*, *PDCDLG2* (PD-L1 and PD-L2, respectively), *CD40*, and *HLA-DRA* (Fig. 3, f and g). The PD1 ligands may be involved in the interaction of FDCs with TFH cells, which express high amounts of PD1 (Crotty, 2019). We hypothesize that these molecules can be used to regulate T cell activity in response to external stimuli. Unexpectedly, *TNFSF13B* (B cell activating factor, BAFF), considered a hallmark FDC protein, was only expressed in MRCs and not in FDCs (Fig. 3 h). Protein expression of BAFF was determined by flow cytometry on permeabilized FDCs and MRCs. The data agreed with the sequencing data and in addition suggested that BAFF on MRCs is membrane-bound since permeabilization was not required (Fig. 3 h). Tonsil sections were stained for RANKL to identify MRCs and the follicle edge. Co-stain with BAFF showed increased signal in MRCs as compared with the follicle, where FDCs reside (Fig. 3 i).

### FDCs express TLRs in abundance

Expression of tlr on mouse FDCs has been shown by immunohistochemistry (tlr4) and functionally in a *tlr7* knock-out mouse model of autoimmunity (Das et al., 2017; El Shikh et al., 2007; Garin et al., 2010). Our scRNaseq data established expression of *TLR4* on human FDCs and demonstrated expression of the adaptor molecules *CD14*, *MYD88*, and *LY96* (MD2), required for TLR4 signaling (Fig. 3 j). *TLR7* expression was not observed in our scRNaseq data but was detected at low levels in our microarray dataset (Fig. S4 a). This dataset correlates highly with our scRNaseq dataset on other aspects (Fig. S1 c). Although *TLR4* was the most dominant, other TLRs were also detected in the scRNaseq data (*TLR1*, *TLR2*, *TLR4*, *TLR5*, and *TLR6*) and in the microarray data (*TLR1*, *TLR4*, *TLR5*, *TLR6*, *TLR7*, and *TLR10*; Fig. S4 a).

### TLR signaling enhances antigen presentation by FDCs

**Figure 4.**
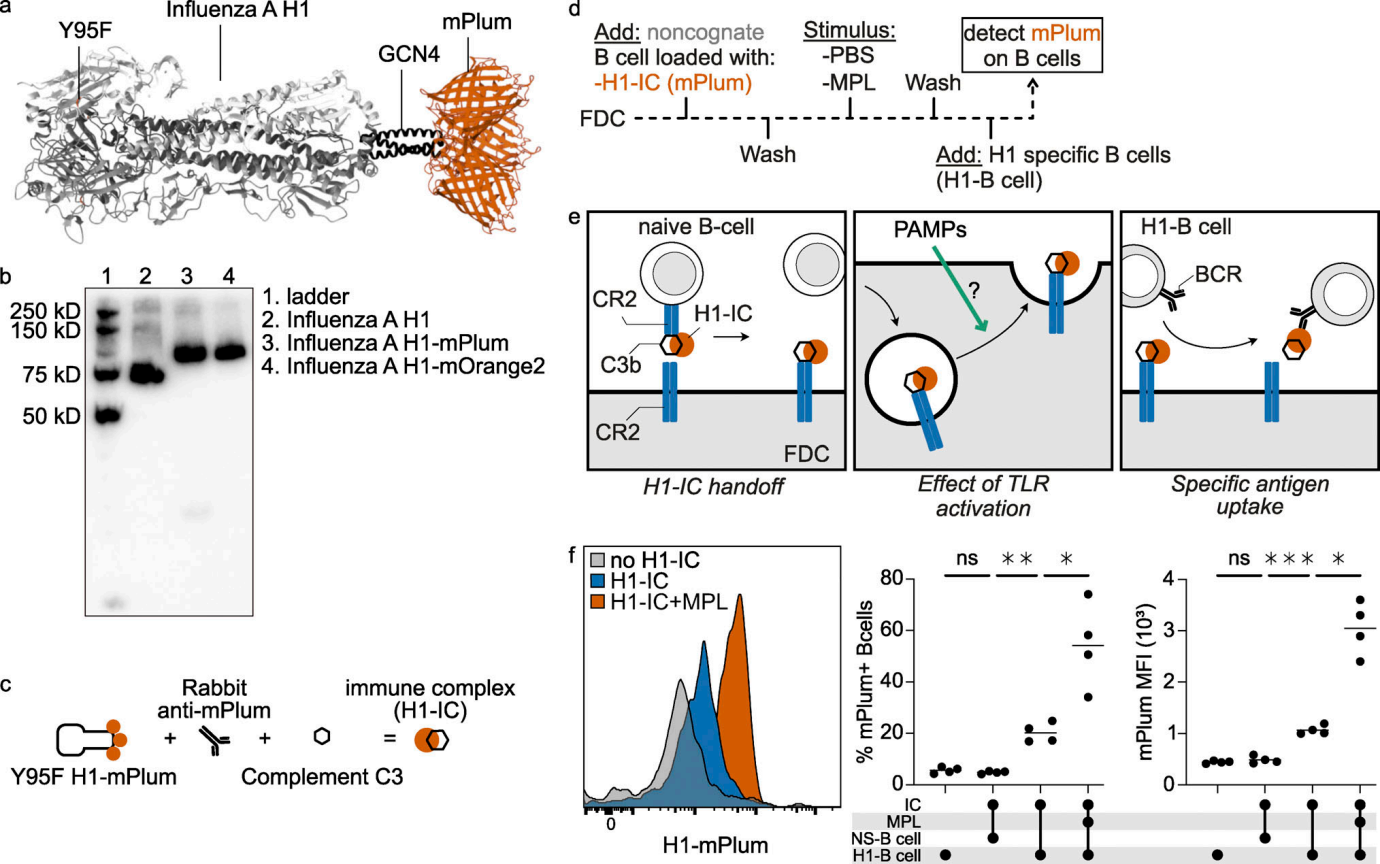
**TLR4 activation on FDCs enhances native antigen presentation.** **(a)** Ribbon representation of H1-mPlum with receptor binding mutant (RBM, Y95F). **(b)** Western blot of influenza H1 protein expressed with and without fluorescent proteins. **(c)** Schematic of IC generation. Ig-depleted serum was used as source of complement; for the IC, negative control heat-inactivated serum was used. **(d)** Timeline of experiment. Sorted cultured FDCs were loaded with influenza H1-IC, washed, and stimulated with MPL or PBS control for 8 h. Medium was replaced, and H1-specific B cells were added for ∼2 h. Then mPlum signal (H1-IC) was measured on H1-specific B cells by flow cytometry. **(e)** Representation of the experiment. H1-IC on B cells was used as a readout for H1-IC available on the FDC surface. **(f)** Histogram of mPlum signal on H1-specific B cells with and without MPL stimulation. No mPlum was detected on B cells when FDCs were not loaded with H1-IC or when a nonspecific B cell line was used (NS-B cell, same donor *S. pneumoniae*–specific). Stimulation with MPL-enhanced specific B cell uptake in percentage as well as MFI. Student’s *t* test or ANOVA, *, P > 0.05; **, P > 0.01; ***, P value > 0.001; *n* = 4 (biological replicates). Related to Fig. S4. PAMP, pathogen-associated molecular pattern.

TLR4 engagement on mouse FDCs induces up-regulation of Fc receptors and integrins, while TLR7 on FDCs promotes auto-reactive B cells in a mouse model of systemic lupus erythematosus through secretion of IFN-α (Das et al., 2017; El Shikh et al., 2007). Because *TLR4* was prominently expressed on human FDCs, the effect of TLR4 activation on antigen presentation to B cells by FDCs was investigated. We made use of the property of FDCs to bind complexes of antigen and complement through CR1 and CR2 and shuttle them to an endocytic recycling compartment. This way, available antigen can be taken up by antigen-specific B cells (Heesters et al., 2013). For these experiments, we used a human B cell clone that was specific for the influenza virus hemagglutinin 1 (H1) and cross-reacted with other group 1 hemagglutinins in a similar fashion to the monoclonal antibody CR6261. The H1-specific B cell clone was generated following immortalization of B cells of an individual vaccinated with an influenza virus vaccine. Following immortalization of peripheral blood B cells by BCL-XL and BCL-6, we single-cell sorted using purified and labeled H1 (Kwakkenbos et al., 2010). These cells were expanded using mouse fibroblasts expressing CD40L in the presence of IL-21 as described previously (Kwakkenbos et al., 2010). The immortalized B cell clone secretes antibody but also highly expresses the B cell receptor on the membrane, making it an excellent tool to measure antigen presentation by FDCs. To generate H1 that was detectable by flow cytometry, we produced a fusion protein of H1 with the RFP mPlum. To disrupt the hemagglutinin binding domain and to prevent binding of H1 to sialic acids on B cells, we generated a Y95F mutant of H1 (Fig. 4 a; and Fig. S4, e–h). H1 bound to H1-specific and -nonspecific B cells through the hemagglutinin binding domain, while the Y95F mutant was only bound by H1-specific B cells (Fig. S4 h). Binding of WT H1 to nonspecific B cells was concentration-dependent and neuraminidase-sensitive, while no binding could be observed with the Y95F mutant (Fig. S4 f). In addition, the use of an extensive glycan array showed negligent binding of the Y95F mutant to glycans and Neu5Acα2-6–specific binding of the WT H1 (Fig. S4 g and Table 1; Nemanichvili et al., 2019; van der Woude et al., 2020). FDCs, purified by flow cytometric cell sorting, were cultured and loaded with complement opsonized ICs of influenza H1 (H1-ICs). ICs were generated by mixing H1-mPlum with an anti-mPlum rabbit antibody and 10% Ig-depleted serum to activate complement (Fig. 4 c). H1-ICs were loaded on noncognate B cells, after which the B cells were washed by centrifugation to remove free H1-ICs and transferred to the FDCs culture. For the mock control H1-ICs, 10% heat-inactivated Ig-depleted serum was used. These ICs did not get opsonized by complement, were not bound by the noncognate B cells, and were thus not transferred to the FDCs (Heesters et al., 2017). Next, the culture was washed to remove B cells and nonbound H1-ICs. We then stimulated FDCs with the TLR4 agonist monophosphoryl lipid A (MPL), a detoxified form of the endotoxin LPS, followed by removal of the agonist and a wash. Then the FDCs were incubated with the H1-specific B cell line (Fig. 4, c and d). Uptake of H1-mPlum by the H1-specific B cell line was analyzed by flow cytometry (Fig. 4, e and f). The fluorescent signal we measured functions as an indirect readout for antigen presentation by FDCs. As controls, FDCs were either not loaded with H1-ICs, or a *Streptococcus pneumoniae*–specific B cell line (NS-B cell), instead of the H1-B cell line, was used for detection. Stimulation of FDCs with MPL resulted in an increase of antigen uptake by specific B cells, in percentage and mean fluorescent intensity (MFI; Fig. 4 f). MPL did not affect the capability of the B cell to take up antigen (Fig. S4 j), indicating that activation of TLR4 on FDCs increased the amount of antigen available for B cells in the GC, presumably by increasing the amount of CR bound H1-IC on the surface. When less antigen was loaded on FDCs, the results remained the same, albeit at lower percentages (Fig. S4 i). Thus, external signals regulate antigen availability by FDCs.

**Table 1.** Glycan array composition

Array no.	Abbreviation	Glycan
1	LN2	Galb1-4GlcNAcb1-3Galb1-4GlcNAcb
2	LN3	Galb1-4GlcNAcb1-3Galb1-4GlcNAcb1-3Galb1-4GlcNAcb
3	NLN1	Galb1-4GlcNAcb1-2Mana1-6(Galb1-4GlcNAcb1-2Mana1-3)Manb1-4GlcNAcb1-4GlcNAcb
4	NLN2	Galb1-4GlcNAcb1-3Galb1-4GlcNAcb1-2Mana1-6(Galb1-4GlcNAcb1-3Galb1-4GlcNAcb1-2Mana1-3)Manb1-4GlcNAcb1-4GlcNAcb
5	NLN3	Galb1-4GlcNAcb1-3Galb1-4GlcNAcb1-3Galb1-4GlcNAcb1-2Mana1-6(Galb1-4GlcNAcb1-3Galb1-4GlcNAcb1-3Galb1-4GlcNAcb1-2Mana1-3)Manb1-4GlcNAcb1-4GlcNAcb
6	3SLN2	Neu5Aca2-3Galb1-4GlcNAcb1-3Galb1-4GlcNAcb
7	3SLN3	Neu5Aca2-3Galb1-4GlcNAcb1-3Galb1-4GlcNAcb1-3Galb1-4GlcNAcb
8	3SNLN1	Neu5Aca2-3Galb1-4GlcNAcb1-2Mana1-6(Neu5Aca2-3Galb1-4GlcNAcb1-2Mana1-3)Manb1-4GlcNAcb1-4GlcNAcb
9	3SNLN2	Neu5Aca2-3Galb1-4GlcNAcb1-3Galb1-4GlcNAcb1-2Mana1-6(Neu5Aca2-3Galb1-4GlcNAcb1-3Galb1-4GlcNAcb1-2Mana1-3)Manb1-4GlcNAcb1-4GlcNAcb
10	3SNLN3	Neu5Aca2-3Galb1-4GlcNAcb1-3Galb1-4GlcNAcb1-3Galb1-4GlcNAcb1-2Mana1-6(Neu5Aca2-3Galb1-4GlcNAcb1-3Galb1-4GlcNAcb1-3Galb1-4GlcNAcb1-2Mana1-3)Manb1-4GlcNAcb1-4GlcNAcb
11	6SLN2	Neu5Aca2-6Galb1-4GlcNAcb1-3Galb1-4GlcNAcb
12	6SLN3	Neu5Aca2-6Galb1-4GlcNAcb1-3Galb1-4GlcNAcb1-3Galb1-4GlcNAcb
13	6SNLN1	Neu5Aca2-6Galb1-4GlcNAcb1-2Mana1-6(Neu5Aca2-6Galb1-4GlcNAcb1-2Mana1-3)Manb1-4GlcNAcb1-4GlcNAcb
14	6SNLN2	Neu5Aca2-6Galb1-4GlcNAcb1-3Galb1-4GlcNAcb1-2Mana1-6(Neu5Aca2-6Galb1-4GlcNAcb1-3Galb1-4GlcNAcb1-2Mana1-3)Manb1-4GlcNAcb1-4GlcNAcb
15	6SNLN3	Neu5Aca2-6Galb1-4GlcNAcb1-3Galb1-4GlcNAcb1-3Galb1-4GlcNAcb1-2Mana1-6(Neu5Aca2-6Galb1-4GlcNAcb1-3Galb1-4GlcNAcb1-3Galb1-4GlcNAcb1-2Mana1-3)Manb1-4GlcNAcb1-4GlcNAcb

### FDCs can interact with T cells through PD-L1 or PD-L2

**Figure 5.**
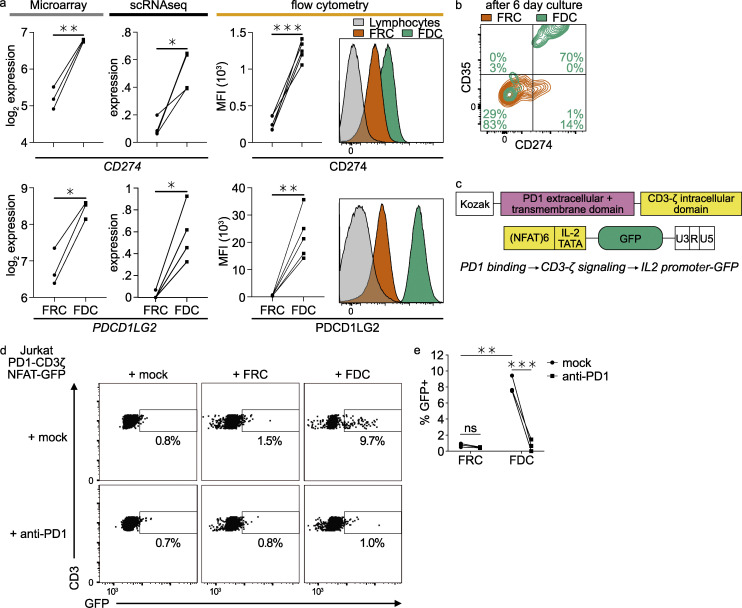
**FDCs express functional PD1 ligands.** **(a)** PD-L1 (CD274) and PD-L2 (PDCD1LG2) gene expression by microarray and scRNaseq and protein expression by flow cytometry. Sctransform-normalized expression. **(b)** Sorted FDCs and FRCs were cultured for 6 d, trypsinized, and analyzed for surface protein expression of CD274 and CD35 by flow cytometry. **(c)** PD1-CD3ζ NFAT-GFP reporter constructs. PD1 activation on the T cell will lead to expression of GFP. GFP, enhanced GFP. **(d)** GFP signal of PD1-CD3ζ NFAT-GFP reporter Jurkat cell line with and without Nivolumab (anti-PD1) after co-culture with FDCs or FRCs. **(e)** Quantification of PD1-CD3ζ NFAT-GFP reporter Jurkat cell line co-culture with FDCs or FRCs. Student’s *t* test or ANOVA, *, P > 0.05; **, P > 0.01; ***, P > 0.001; scRNaseq data, *n* = 4; microarray data, *n* = 3; flow cytometry, *n* = 5; Jurkat reporter data, *n* = 3 (biological replicates). Related to Fig. S5.

**Figure S5.**
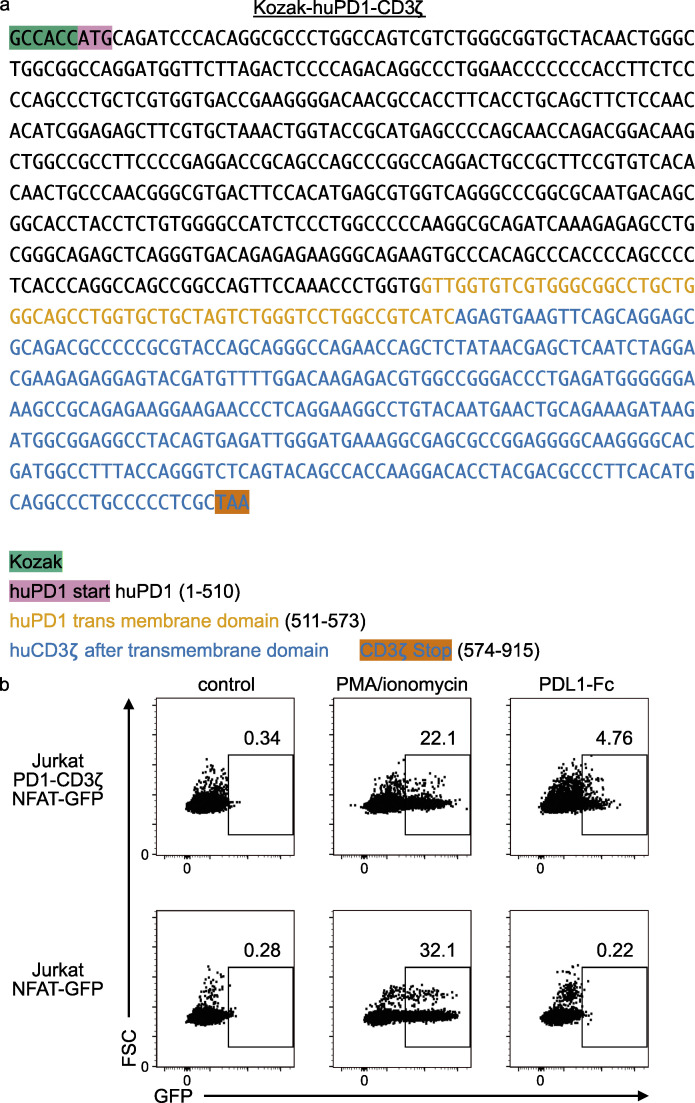
**Jurkat human PD1-CD3ζ NFAT-GFP reporter.** **(a)** Kozak-huPD1-CD3zeta sequence. The human PD1 extracellular and transmembrane domain were coupled to the human CD3ζ signaling domain (everything after the transmembrane domain). **(b)** Jurkat PD1-CD3ζ NFAT-GFP reporter and NFAT-GFP reporter control. PMA/ionomycin stimulation results in a maximum GFP signal of 20–30%, while PD-L1–Fc only achieves 5% GFP-positive cells. The system is sub-optimal but can detect PD1–PD-L1 interaction.

Expression of significant levels of *CD274* (PD-L1) and *PDCD1LG2* (PD-L2) on FDCs has not been observed previously. Gene expression was shown by microarray and scRNaseq (Fig. 5 a). Flow cytometry showed significant protein expression of PD-L1 and PD-L2 on the FDC surface (Fig. 5 a). In vivo, PD-L2 protein was shown in frozen tissue sections by fluorescent microscopy (Fig. 2 b). To see if these proteins were functional, we assessed interaction with PD1. Sorted FDCs still expressed PD-L1 and CD35 after a 6-d culture, while FRCs hardly up-regulated PD-L1 (Fig. 5 b). A Jurkat PD1-binding reporter T cell line in which the binding of PD1 results in expression of GFP was produced. The PD1 extracellular domain was coupled to the CD3ζ intracellular domain, and GFP was expressed behind the enhanced IL-2 promoter, resulting in GFP expression upon PD1 activation (Fig. 5 c; and Fig. S5, a and b). Co-cultures with FDCs induced GFP expression, while co-cultures with FRCs did not (Fig. 5 d). Detected GFP levels after co-culture with FDCs were higher than induced by soluble PD-L1-Fc. The expression of GFP could be blocked by preincubation of the T cells with the anti-PD1 monoclonal antibody nivolumab, as expected (Fig. 5 e). These data show that PD-L1 and PD-L2 are present on FDCs and that these proteins can functionally activate PD1 on Jurkat T cells.

## Discussion

Here we present an extensive characterization of human FDCs. We show that FDCs are more than antigen depots and form a functional regulatory unit within the GC. Our data reveal that FDCs express TLRs, which can sense pathogen-associated molecular patterns. Furthermore, our data suggest that FDCs can regulate T cell activity through PD1 ligand interactions. These data find their basis in scRNaseq analyses and are further strengthened by functional experiments. This work provides transcriptomic data as a resource for future studies, human FDC markers, sorting strategies, an improved culture method for sorted human FDCs and functional data on human FDCs.

An integrated comparison of our data with data collected in a study in mice (Rodda et al., 2018), converted to human homologous genes revealed that human and mouse FDCs cluster together, and many expressed genes were conserved across species. But we also found differences between mouse and human FDCs. Human and mouse FDCs expressed *CXCL13*, *CR2*, *MFGE8*, *FCER2*, *SOX9*, and *TMEM119*, although the latter two are not only expressed by FDCs in humans as they were also found in MRCs. Compared with mouse, human FDCs express more *FCER1G*, *FCAMR*, *CD52*, and *SERPINE2*. The differences we find can be species-specific, inflammatory state–dependent, or location-dependent. Rodda et al. (2018) collected the mouse data used here from both naive and inflamed lymph nodes, used all skin-draining lymph nodes of multiple mice, and pooled the data to get enough FDCs. In contrast, we acquired the data on human FDCs from tonsils that were at least partly inflammatory. We included multiple donors while retaining individual resolution through index sorting. Although both studies find a homogeneous population of FDCs, which correlates well with in situ microscopy data, it cannot be ruled out that tissue processing introduced selection biases. This is a known problem for all studies that use mechanical or enzymatic force to disrupt tissues (Denisenko et al., 2020).

The highly expressed complement receptors perform two of the major functions of FDCs, retention and presentation of ICs (Heesters et al., 2016; Roozendaal and Carroll, 2007). In mice, Cr1 and Cr2 are produced by the *Cr2* locus via alternative mRNA splicing, while in humans, CR1 and CR2 are encoded by separate genes. This complicates the interpretation of experimental data on CR function in FDCs. In mice, Cr1 and Cr2 have the same C terminus and share a complement binding site, in contrast with humans. *CR1 *and* CR2* are highly expressed by human FDCs and enable the binding of C4b and C3b complexes by CR1 and (i)C3d(g) complexes by CR2 (Heesters et al., 2014; van Nierop and de Groot, 2002). It is interesting that despite the differential regulation, humans and mice both express the two receptors on FDC. The data presented here show a significant expression of CR1 and an even higher expression of CR2.

CR1 can bind C3b and facilitate the conversion to the more stable iC3b (by factor I) and C3d, which CR2 can bind. Binding of complement C4 ICs could follow a similar mechanism, where again CR1 can bind C4b and facilitate the conversion to the more stable iC4b and C4d, which CR1L can bind (Logar et al., 2004). So it seems a reasonable hypothesis that FDCs bind C3b and C4b IC through CR1 first and, after stabilization of the IC, through CR2 and CR1L, respectively. This would argue that long-term retention of ICs is CR2- and CR1L-mediated. FDCS might also be able to distinguish ICs opsonized with C3 from those opsonized with C4.

All this complement activity at the FDC surface requires protection from the lytic complement pathway. CD55 could mediate this and was expressed more on FDCs compared with other cell types. Another functionality of CD55 could be the prevention of bacterial IC lysis and thus of antibody production against internal epitopes. Data on endocytic retention of intact bacteria by FDCs support this idea (Heesters and Carroll, 2016).

FDCs also expressed *C5AR1*, which can detect C5a concentration. This opsonin is a measure of inflammation. Together, FDCs express a plethora of complement-associated proteins that can bind, regulate, and sense the complement system.

Besides complement receptors, Fc receptors can bind the antibody part of ICs. Ex vivo IC-trapping experiments in mice showed some limited trapping of ICs in the absence of serum (complement), which was blocked by anti-FcγRIIβ antibodies (Yoshida et al., 1993). Mice with FcγRIIβ-deficient FDCs had unaltered primary antibody responses, but impaired recall responses (Qin et al., 2000). Yet in the human FDC data, no expression of *FCGR2B* was detected. Instead, *FCER2* (CD23), *FCAMR*, and *FCGRT* were expressed, in contrast with transcriptional data of mouse FDCs (Rodda et al., 2018). FCER2 (CD23), the low-affinity receptor for IgE, is known for its role in antibody feedback regulation in B cells. FCAMR binds IgM with high affinity and IgA with a 10-fold lower affinity, while FCGRT, the neonatal Fc receptor, monitors IgG turnover and is reported to clear IgG and albumin from the liver (Pyzik et al., 2017). In combination with the limited effects on IC trapping, this would argue for a different role of Fc receptors on FDCs. A good candidate is perception of the environment, which could lead to regulation. Fc receptors on human FDCs perhaps sense the predominant Ig in the GC and could steer class switching in the GC. This is in line with the subtle FcR signaling in other cell types. In human classic DCs, for instance, FcR engagement enhanced crosstalk with TLRs and skewed cytokine production toward a more pro–T helper type 17 cell profile (den Dunnen et al., 2012).

Our data show that BAFF (*TNFSF13B*) was not expressed by FDCs but by MRCs. This is in striking contrast with the literature claiming BAFF expression on FDCs. Many reviews proposed the secretion of BAFF by FDCs as the selection mechanism for GC B cells. However, direct evidence to support BAFF expression by FDCs was lacking and solely based on imaging of the follicle and qPCR of impure cell suspensions (Hase et al., 2004). A recent study showed the BAFF protein to be present in all regions of the follicle, with the highest concentration in the mantle zone, a region where MRCs reside (Carrillo-Ballesteros et al., 2019). This is consistent with our expression data, and together with recent literature, this supports an outward-in gradient instead of an inward-out gradient of BAFF in the follicles, designating MRCs and not FDCs as the major BAFF producers.

These data are significant because they challenge the dogma that B cells that interact stronger and longer with FDCs due to a high-affinity B cell receptor receive more survival signals in the form of BAFF from the FDCs. Instead, our data support a model in which the surrounding MRCs provide BAFF to the entire follicle.

Our study shows the expression of multiple TLRs by human FDCs and a regulatory mechanism for antigen presentation in response to TLR activation. This is in line with earlier studies in mice that showed up-regulation of Fc receptors and integrins after TLR4 stimulation (El Shikh et al., 2007). Activation of TLR4 can trigger a MYD88-dependent or MYD88-independent pathway, which in turn can activate NF-κB or IRF3 pathways, respectively. Little is known about signaling cascades in FDCs, but in other cell types, PD-L1 and PD-L2 expression is induced by NF-κB through type I IFNs (Sun et al., 2018; Asgarova et al., 2018). The interaction of PD-L1 with PD1 on T cells is famous for the success of PD1 blocking antibodies as anti-tumor drugs, but this interaction is difficult to study in vitro. Tumor cells express PD-L1 to inhibit T cell activity through PD1, and anti-PD1 therapy removes this brake, which activates the T cells. This interaction has physiological relevance, but details on homeostatic conditions are lacking. Recently we established a technology to generate GC B cell clones with defined specificity (Kwakkenbos et al., 2010, 2016; Nojima et al., 2011). Using a clone specific for group 1 influenza virus hemagglutinin (H1), we demonstrate that stimulation with TLR4 agonist greatly enhanced the capacity of FDCs to present antigen to the B cells. TLR agonists are known to enhance the potency of vaccines. Our data clearly indicate that TLR4 in vaccines not only may increase APC activities of hematopoietic dendritic cells (Higgins et al., 2006; Jang et al., 2021) but also has a powerful stimulating effect on FDC antigen presentation to B cells in GCs. Pathogen-associated molecular patterns <70 kD should be able to pass through the conduit system and enter the GC. Electron microscopy studies suggest a direct connection between conduits and FDCs (Gonzalez et al., 2011). TLR4 activation leads to more transferrin receptor on the cell surface in dendritic cells (Burzyn et al., 2004). Incidentally, recycling endosomes containing ICs in FDCs colocalize with transferrin receptor, which is also recycled to the cell surface (Heesters et al., 2015; Mayle et al., 2012). This suggests a potential mechanism for regulation of receptor availability by TLR4. Our data confirm that FDCs can present antigen to B cells, but also strongly suggest that FDCs interact with T cells. Expression of class II MHC molecules indicates that FDCs can present antigens to T cells. FDCs may also interact with PD1^+^ TFH cells through PD-L1 and PD-L2. FDCs also express CD73, which mediates hydrolysis of ADP and ATP to adenosine, dampening danger signals. CD200 on FDCs can deliver inhibitory signals to immune cells, including T and B cells, through CD200R. Thus, FDCs may have negative regulatory functions on T cells, particularly TFH cells. However, the fact that FDCs also expressed CD40 may imply that these cells may also positively stimulate T cells. Considering the data, the role FDCs play in the B cell follicle may need revision. The dogma that FDCs merely hold antigen and provide BAFF to the strongest and longest interactors for B cell survival is not in line with this study or other recent studies that show selection of lower-affinity B cell clones (Viant et al., 2020). TFH cells clearly play an important role in the selection of B cells in the GC. PD1^+^CXCR5^+^ TFH cells accumulate in the follicle during GC formation, are not clonally restricted, and can be exchanged between follicles, unlike GC B cells (Shulman et al., 2013). The expression of PD-L1, PD-L2, and HLA-DR together with the secretion of CXCL13 make FDCs ideal partners for TFH cells. The interaction of TFH cells with FDCs was observed in HIV-infected individuals (Dave et al., 2018; Heesters et al., 2015).

In conventional DCs, PD-L2 is up-regulated upon stimulation with IL-4, which is produced by TFH in the GC (Lesterhuis et al., 2011; Vijayanand et al., 2012). FDCs also express the common γ-chain and its partners IL-4R and, to a lesser extent, IL-15R. Thus, they may be able to respond to IL-4 and IL-15. The interaction strength between TFH and GC B cells governs whether the B cell becomes a plasma cell or recycles as a GC B cell (Ise et al., 2018). We propose FDCs have a regulatory role in the selection process of B and T cells by interacting with both. FDCs have the machinery to interact with and regulate B cells and T cells. It is very well possible this interaction takes place at the same time, forming a triad of T cell, B cell, and FDC. This would allow for regulatory control.

A recent study found that, not unlike GC B cells, TFH cells undergo selection in the GC in an antigen-dependent matter (Merkenschlager et al., 2021). The expression of HLA-DR by FDCs could provide a mechanism of matched T and B cell selection at a single site. Not all antigen taken up by FDCs is shuttled to a recycling endosomal compartment; a significant portion is transferred to degradative compartments (Heesters et al., 2013). Since FDCs are known for their presentation to B cells, this was considered a mechanism to clean up excess antigen. This also opens the possibility for presentation of peptides by HLA molecules in a GC to select TFH.

In conclusion, FDCs have the machinery to play a role in regulation of the GC, but how they play this role needs to be elucidated in future studies. Collectively, our results indicate that FDCs express many regulatory proteins for both B and T cells. Moreover, FDCs can sense their environment through TLRs and act accordingly by modifying antigen presentation.

## Materials and methods

### Stromal cell isolation and cell sorting

Stromal cells were isolated from discarded palatine tonsils from routine tonsillectomies. The collection and use of all human samples was approved by the Medical Ethical Committee of the Amsterdam Medical Center and with informed consent. Stromal cell isolation was modified from that described (Heesters et al., 2017). Tonsils were harvested into HBSS (Gibco) with 2% FBS (GE Healthcare), 10 mM Hepes (Gibco) and penicillin/streptomycin (P/S; Roche) on ice. Tissue was cut into thin slices and strained through a metal grid (1 mm^2^) with a glass pestle. Supernatant was aspirated and tissue chunks transferred to digestion buffer for 10 min (HBSS, 0.5% FBS, 15 mM Hepes, 1 mM sodium pyruvate [Gibco], P/S, 0.6 mg/ml collagenase D [Roche], and 0.5 mg/ml DNase I [Roche]). Supernatant was moved to HBSS with 2% FBS and 2 mM EDTA on ice. Remaining tissue pieces were incubated with digestion buffer three more times. Tissue was triturated using Pasteur pipettes of decreasing sizes between digestions. Single-cell suspension was then filtered and underlaid with 1.03 g/ml and 1.045 g/ml Percoll centrifugation media (GE Healthcare) in PBS. This equals 21% and 34% standard isotonic Percoll, respectively. Interphase was collected, washed, and stained with anti-CD31 BV421 (WM59), anti-CD45 BV421 (2D1), anti-CD35 FITC (E11), PDPN PE/Cy7 (NC-08), and helix-NP (nuclear probe) viability marker (APC) in HBSS (plus 2% FBS, 20 mM Hepes, 2 mM EDTA, 1 mM sodium pyruvate, and P/S). All antibodies were from BioLegend, unless specified otherwise. Viable, CD45^−^CD31^−^ cells were sorted on a low-pressure cell sorter (Sony SH800S) with a 100-µm nozzle. Cells were sorted in bulk or into 384-well plates for subsequent CEL-Seq2 sequencing (Single Cell Discoveries). Cells with the lowest helix-NP staining were excluded as SSC-A^low^CD45^−^CD31^−^PDPN^−^ insufficiently stained CD45^+^ cells. FDCs were further sorted as PDPN^+^CD35^high^. For culture, cells were sorted in culture media supplemented with 20% FCS.

### Flow cytometry analysis

For validation of individual markers, cells were analyzed on a Fortessa LSR (BD Biosciences). Stromal cells were isolated as above and stained additionally with (antibody [clone]) anti-CD21 (Bu32), anti-CD137 (4B4-1), anti-CD55 (JS11), anti-CD49e (NKI-SAM-1), anti-TLR4 (HTA-125), anti-CD73 (AD2), anti-CD200 (OX-104), anti-CD23 (EBV-CS5), anti-CD273 (24F.10C12), anti-CD274 (29E.2A3), anti-CD171 (L1-OV198.5), anti-CD14 (63D3), anti-CD106 (A16047A), anti-CD104 (58XB4), anti-CD11B (ICRF44), anti-CD40 (5C3), and anti-HLA-DR (L243). All antibodies were from BioLegend, unless specified otherwise. Data were analyzed using FlowJo (TreeStar) and Prism (GraphPad).

### CEL-Seq2–based single-cell RNA sequencing

Cells were directly sorted in 384-well plates, frozen, and sent to Single Cell Discoveries for further processing of their pipeline based on CEL-Seq2 (Hashimshony et al., 2016; Muraro et al., 2016). Libraries were run on a HiSeq4000 for Illumina sequencing. Post-processing and quality control were performed by Single Cell Discoveries. Reads were aligned to GRCh38 reference assembly using STARsolo (Dobin et al., 2013). Primary assessment reported 15,235 median unique molecular identifiers (transcripts) per cell and 4,833 median genes per cell sequenced to 85.7% sequencing saturation with 180,108 mean reads per cell.

### Microarray

To isolate total RNA, sorted cells were flash-frozen in PBS immediately after sorting and stored at −80°C before RNA extraction. QIAzol Lysis Reagent (Qiagen) was added to the cells, and RNA was isolated and purified using the RNeasy kit (Qiagen). The concentration was measured on a NanoDrop ND-2000 (Thermo Fisher Scientific), and RNA integrity was examined using the 2200 TapeStation System with Agilent RNA ScreenTapes (Agilent Technologies). Total RNA was amplified using the GeneChip WT Pico Kit (Thermo Fisher Scientific) generating biotinylated sense-strand DNA targets. The labeled samples were hybridized to human Clariom S pico arrays (Thermo Fisher Scientific). Washing and staining were performed using the GeneChip Fluidics Station 450, and scanning was performed using the GeneChip Scanner 3000 (both Thermo Fisher Scientific). All cell populations were generated in triplicate.

### Data analysis

All transcriptome data analysis was performed in RStudio running R. For the scRNaseq, the Seurat package was used in combination with the tidyverse package. For the microarray, raw data were normalized using the robust multiarray average algorithm implemented in the limma package. Adjusted P values were calculated using the Benjamini–Hochberg method. Data were visualized using Seurat v4 and tidyverse R packages (Butler et al., 2018; Hao et al., 2020 *Preprint*; R Core Team, 2020; Ritchie et al., 2015; Satija et al., 2015; Stuart et al., 2019; Wickham et al., 2019).

Raw single-cell RNA sequencing data and microarray data are deposited in GEO under accession no. GSE178272. Mouse data from Rodda et al. (2018) are deposited in GEO under accession no. GSE112903. Processed data are available as a webtool at https://heesterslab.shinyapps.io/FDCshiny/.

### Cell culture

Sorted FDCs were resuspended in FDC media (IMDM, 5% FCS, and 1× nonessential amino acids, 1× sodium pyruvate, 10 µg/ml gentamicin, 10 ng/ml NGF, and 10 ng/ml FGF) and plated on tissue culture–treated 96-well flat bottom plates. 5–7-d cultured FDCs were used for different assays.

### mPlum H1 ICs and H1-specific B cells

H1 sequence originating from A/Puerto Rico/8/1934/H1N1 (PR8 Cambridge strain; GenBank accession no. NP_040980) and was previously described in Nemanichvili et al. (2019). pCD5-PR8-GCN4-TEV-mPlum plasmid encodes for a GCN4 leucine zipper trimerization motif (Harbury et al., 1993), followed by a 7–amino acid cleavage recognition sequence (ENLYFQG) of tobacco etch virus (TEV), an mPlum fused to H1, and a strep-tag II (WSHPQFEKGGGSGGGSWSHPQFEK; IBA) C-terminally. To avoid sialic acid binding to B cells, the Y95F mutation was introduced using the Q5 site-directed mutagenesis kit.

pCD5-H1^−^ with or without GCN4-mPlum expression vectors was transfected into HEK293S GNTI(−) cells (modified HEK293S cells lacking glucosaminyl transferase I activity; CRL-3022; American Type Culture Collection) with polyethyleneimine I in a 1:8 ratio (µg DNA:µg polyethyleneimine I) as previously described (de Vries et al., 2010). The transfection mix was replaced after 6 h by serum free media (293 SFM II suspension medium, 11686029; Invitrogen; supplemented with glucose 2.0 g/l, sodium bicarbonate 3.6 g/l, primatone 3.0 g/l [Kerry], 1% GlutaMAX [Gibco], 1.5% DMSO, and 2 mM valproic acid). Culture supernatants were harvested 5 d after transfection. The H1 expression was analyzed with SDS-PAGE followed by Western blot on polyvinylidene fluoride membrane (BioRad) using α-strep-tag mouse antibodies 1:3,000 (IBA Life Sciences). Subsequently, H1 proteins were purified with Sepharose Strep-Tactin beads (IBA Life Sciences) as previously described (de Vries et al., 2010).

mPlum-ER-3 was a gift from Michael Davidson (plasmid #55966; Addgene; http://n2t.net/addgene:55966; RRID:Addgene_55966).

H1 ICs were generated by mixing 1 µg HA1-mPlum, 0.5 µg rabbit anti-RFP IgG (Abcam), and 10% human serum (from an HA1-naive individual, depleted for IgG, as a source of complement) in GVB^++^ buffer (Gelatin Veronal Buffer with Ca and Mg; in-house) for 30 min at 37°C. One million Raji cells were then incubated with the IC mix for 30 min at 37°C to generate B cells with ICs bound through CR2. Free ICs were washed away, and Raji cells were added to the FDC culture for 2 h at 37°C. Raji cells were washed away before stimulation or addition of H1-specific B cells. The H1-specific B cell line (AT10-005) was generated as described and has a specificity identical to CR6261 (Kwakkenbos et al., 2010). Then H1-specific B cells were aspirated for further measurement of mPlum signal on a Fortessa LSR (BD Biosciences) to assess IC binding.

### PD1-GFP reporter Jurkat cell line

The PD1 extracellular domain was cloned and coupled to the CD3ζ intracellular immunoreceptor tyrosine-based activation motif domain. The engineered receptor contains a Kozak sequence, a human PD1 extracellular domain, a human PD1 transmembrane domain, and the human CD3ζ intracellular immunoreceptor tyrosine-based activation motif domain after the transmembrane domain. Activation of PD1 results in downstream activation of NFAT and the IL-2 promoter, a well-characterized signaling pathway (Fathman and Lineberry, 2007). This activates the self-inactivating–(NFAT)x-GFP reporter construct, resulting in GFP expression (Hooijberg et al., 2000). The sequences of the Kozak-huPD1-CD3ζ reporter construct and controls are provided as supplemental data (Fig. S5).

### Fluorescent microscopy

Tissue samples were frozen in Cryomatrix (37288; Thermo Fisher Scientific) at −80°C. Cryostat sections (4 µm thick) were cut at −25°C on a Cryostar NX70 cryostat (Thermo Fisher Scientific) and picked up on glass slides (KP-Silan Printer microscope slides, adhesive; PR-S-001). Freshly cut cryostat sections were encircled with a paraffin adhesive pen (Dako) and air-dried at room temperature (RT) for 1 h before immunohistochemical staining or stored at −80°C until use. Sections were fixed in acetone (−20°C) for 10 min and washed three times with wash buffer (PBS with 0.05% Tween-20). Sections were incubated with blocking buffer (PBS with 0.05% Tween-20 and 2% BSA) for 30 min and stained with primary antibodies diluted in PBS with 10% blocking buffer for 1 h. If a subsequent antibody staining was desired, slides were washed three times with wash buffer and stained with secondary antibodies diluted in PBS with 10% blocking buffer for 1 h. Last, slides were washed twice with wash buffer and once with PBS containing Hoechst. Slides were washed once more with PBS and mounted using Prolong antifade (P36934). Slides were stored at 4°C. Imaging was performed on a Leica DMI6000 inverted microscope. Images were analyzed using Icy (de Chaumont et al., 2012). For the BAFF staining, formalin-fixed, paraffin-embedded human tissue sections 3 µm in thickness were treated in xylene, a decreasing alcohol gradient, and distilled water to achieve de-waxing and rehydration of the tissue. Heat-induced epitope retrieval was performed for 15 min in Tris-EDTA buffer, pH 9. After epitope retrieval, tissue sections were permeabilized with 0.2% Triton X-100 in PBS, blocked with 5% BSA and 5% Fc receptor blocking (Miltenyi Biotec), and stained with rabbit polyclonal anti-human BAFF (Abcam) and mouse anti-human RANKL (receptor activator of NF-κΒ ligand; Thermo Fisher Scientific), followed by fluorochrome-conjugated donkey secondary antibodies (Jackson ImmunoResearch). Nuclear DNA was visualized with DAPI and coverslips applied with FluorSave reagent (Merck Millipore). Images were acquired with a Nikon Eclipse Ni-E microscope (Nikon) and were further analyzed with ImageJ software.

### Online supplemental material

Fig. S1 provides details on the microarray data acquisition and on the correlation of the human scRNaseq data with the human microarray data or the mouse scRNaseq data published in 2018. In Fig. S2, the highest expressed genes by scRNaseq and microarray in FDCs are highlighted and visualized in heatmaps. Fig. S3 provides volcano plots comparing FDCs, FRCs, and MRCs. Additional microscopy and Pearson’s correlation for the measured proteins with CD35 are shown. Fig. S4 provides details on pathways enriched in FDCs and matches the scRNaseq data with the microarray data. Furthermore, this figure contains experiments that validate the binding of H1 to specific glycans and the loss of this binding in the Y95F mutant H1. In Fig. S4, the effect of MPL on H1 binding by H1-specific B cells and binding of H1 and the Y95F mutant to these B cells are shown. Fig. S5 provides the sequence of the Kozak-huPD1-CD3ζ reporter construct and controls for the resulting reporter cell line.

Additionally, a webtool is available at https://heesterslab.shinyapps.io/FDCshiny/, where genes of interest can be queried for expression by FDCs.
